# Photoacoustic needle improves needle tip visibility during deep peripheral nerve block

**DOI:** 10.1038/s41598-021-87777-9

**Published:** 2021-04-19

**Authors:** Kunitaro Watanabe, Joho Tokumine, Alan Kawarai Lefor, Harumasa Nakazawa, Katsuya Yamamoto, Hiroyuki Karasawa, Miki Nagase, Tomoko Yorozu

**Affiliations:** 1 Department of Anesthesiology, Kyorin University School of Medicine, 6-20-2 Shinkawa, Mitaka City, Tokyo 181-8611 Japan; 2 Department of Surgery, Jichi Medical University, Shimotsuke, Tochigi Japan; 3 Medical System Research & Development Center, FUJIFILM Corporation, Kanagawa, Japan; 4 Department of Anatomy, Kyorin University Faculty of Medicine, Mitaka, Tokyo Japan

**Keywords:** Nervous system, Neurology, Risk factors

## Abstract

We developed a novel technology using the photoacoustic effect that improve needle tip visibility. We evaluated whether this technology improves needle tip visibility when performing a deep peripheral nerve block in a cadaver model. A photoacoustic needle was developed using a conventional echogenic needle with an intraluminal optical fiber. A pulsed laser sends light from a source through the fiber, which is converted to ultrasound at the needle tip using the photoacoustic effect. A nerve block expert performed deep nerve blocks using the photoacoustic needle and the ultrasound views recorded, with or without photoacoustic ultrasound at the needle tip. Needle tip visibility was evaluated by questionnaire (Likert scale 1: very poor, 5: very good) completed by anesthesiologists evaluating recorded images. The score was presented as median [first quartile, third quartile]. Statistical analysis was performed using the Wilcoxon matched-pairs signed rank test. The scores of needle tip visibility with photoacoustic ultrasound from the needle tip (4.3 [4.0, 4.5]) was significantly higher than that without photoacoustic ultrasound (3.5 [3.2, 3.8]) (p < 0.01). Ultrasound emitted at the needle tip using the photoacoustic effect improves needle tip visibility during deep peripheral nerve blocks.

**Clinical trial number** University Hospital Medical Information Network Center Clinical Trials Registration System (UMIN000036974).

## Introduction

Ultrasound-guided peripheral nerve block of deep targets remains a challenge in anesthesiology^1^. Needle trajectory tends to be steep to approach deep neural targets. The needle tip is difficult to image because the reflection of sound waves to the probe from the needle is reduced by the steep angle^2,3^. Therefore, deep nerve blocks are technically difficult, and considered as high-risk procedures^1^.

The photoacoustic effect was discovered in 1880 by Alexander Graham Bell^4^. Clinical applications of this technology have been tried^5^. Piras et al. first reported ultrasound-emission from a biopsy needle using the photoacoustic effect^6^. In their report, the optical fiber was inserted into a biopsy needle for guidance, and then was then removed during the biopsy. Kang also reported a photoacoustic biopsy needle^7^. We developed a photoacoustic needle with a technological innovations (2017)^8^, allowing use of a smaller caliber needle, a flexible optical fiber, and less invasive (the light source generates two microjoules). A novel photoacoustic needle for ultrasound-guided peripheral nerve block was developed for this study.

We evaluated whether the ultrasound-emitting needle tip improved needle tip visibility compared to a conventional echogenic needle during ultrasound-guided deep nerve blocks in a cadaver model.

## Materials and methods

This study was reviewed and approved by the local ethics committee (Kyorin University Ethical Review Board, Reception No. 1408, approved date 2019.12.6) and registered in the University Hospital Medical Information Network Center Clinical Trials Registration System (UMIN000036974). The study was conducted in accordance with Consolidated Standards of Reporting Trials (CONSORT) guidelines (Supplemental information; “CONSORT Diagram”).

The cadaver used in this study was donated to Kyorin University School of Medicine for anatomical education, research, and clinical skills training. The research protocol was prepared in strict accordance with the "Guidelines for the research involving cadavers" of the Japanese Association of Anatomists, and was approved by the Ethics Committee of Kyorin University Faculty of Medicine (Permit No. 986). A comprehensive consent form was obtained from the donor prior to donation and from the family prior to and at the time of donation. The research outline was published on the Kyorin University website to ensure that the families had the opportunity to refuse (opt-out).

### Ultrasound-emitting from needle tip using photoacoustic effect

**Figure 1:**
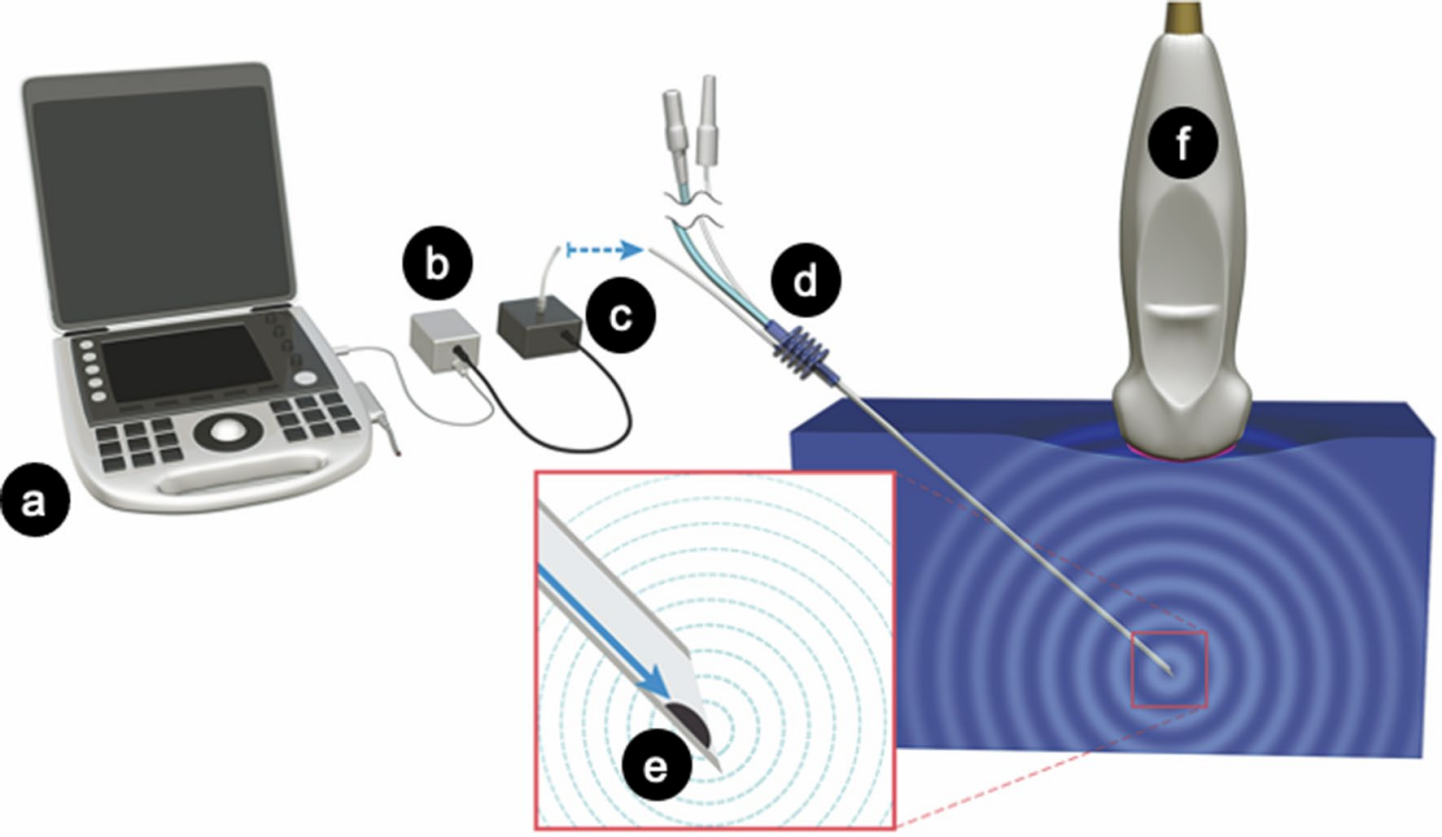
Configuration of the system for photoacoustic needle. Ultrasound machine (**a**) supplies power to an output regulation port (**b**), which controls timing of electric power for generating laser light (**c**). The laser is transmitted through the optical fiber integrated in the lumen of the needle (**d**). The black resin (**e**) absorbs the light, and then converts it to ultrasound by a photoacoustic effect. The ultrasound probe detects the photoacoustic ultrasound. (**a**) Ultrasound machine, (**b**) electrical output regulation port, (**c**) laser light source, (**d**) photoacoustic needle, (**e**) black resin, (**f**) ultrasound probe.

The principle of an ultrasound-emitting system using a photoacoustic effect is described (Fig. 1)^8^. An optical fiber is inserted into the lumen of a needle, and fixed in place maintaining the lumen open for administration of local anesthetic agents. The fiber tip is covered with black resin containing carbon-black pigment, and fixed to the inside wall at the needle bevel. Pulsed laser from an external laser light source is transmitted through the optical fiber. The black resin at the needle tip absorbs the pulsed laser light, which causes adiabatic thermal expansion, and is translated to high frequency vibrations. As a result, ultrasound waves are generated by the photoacoustic effect. The ultrasound wave is received by the ultrasound transducer in the ultrasound probe, which is converted into electrical signals and transferred to the ultrasound unit for imaging. Typical ultrasound imaging for scanning tissue involves a “round trip” for the ultrasound waves, emitted from the transducer in the probe and reflected by the needle back to the probe. However, the ultrasound wave generated by the photoacoustic effect is a one-way trip, emitted at the needle tip to the probe. The ultrasound frame to create a view for the ultrasound wave generated by the photoacoustic effect needs 11% of all frames. This reduction in the frame rate did not inhibit smooth dynamic ultrasound views in the preliminary study. Safety evaluation of the system was proven in a previous study according to the “Safety of laser products—Part 1: Equipment classification and requirements: IEC60825-1:2014”^8^. The ultrasound waves generated by the photoacoustic effect is colored green or white.

**Figure 2:**
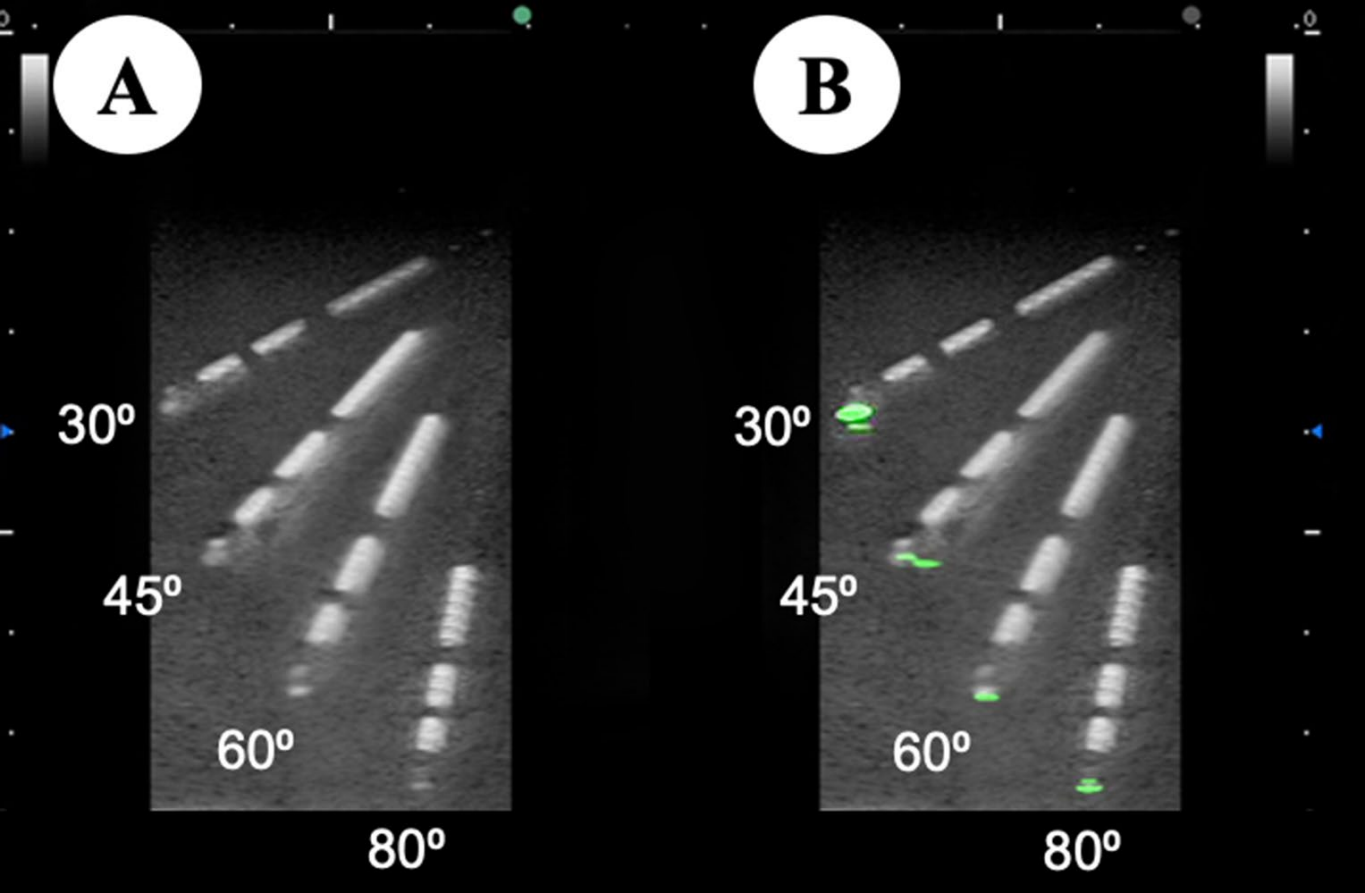
Needle tip visibility at several trajectories. Stimuplex Ultra 360 (B. Braun Medical Inc., Melsungen, Germany) and a photoacoustic needle (an optical fiber is incorporated into Stimuplex Ultra 360) are inserted into UGP GEL (main ingredient; 20% Agar, Alfabio Co., Gunma, Japan) at trajectories from 30° to 80°. Although needle shafts of both needles are always clearly visible at any trajectory, needle tip visibility of the conventional needle tip gradually decreases with an increased trajectory angle. However, the needle tip of the photoacoustic needle can be identified clearly even at 80°. (**A**) Stimuplex Ultra 360, (**B**) Photoacoustic needle.

In the study, we used an ultrasound machine FC1 (FUJIFILM Medical Co., Ltd., Tokyo, Japan) and ultrasound probe L38xp/13-6 and C35xp/8-3 (FUJIFILM SonoSite, Inc., Bothell, WA, USA). The nerve block needle was a Stimuplex Ultra 360 (insulated echogenic needle; size 22 G, length 80 mm, bevel angle 30°, B. Braun Medical Inc., Melsungen, Germany), in which an optical fiber was incorporated as described above). Figure 2 shows ultrasound views at several trajectory needle angles of the echogenic needle or ultrasound-emitting needle using the simulator.

### Ultrasound-guided deep nerve block

An expert (K.W.) in ultrasound-guided nerve blocks performed these nerve blocks on a cadaver under ultrasound guidance using the novel ultrasound-emitting needle. The cadaver (an 85-year-old man fixed with N-vinyl-2-pyrrolidone) had soft and pliable tissue^9^. The deep nerve blocks performed included a paravertebral block (transversal technique with lateral to medial direction, in-plane approach), lumbar plexus block (transverse technique with lateral to medial direction, in-plane approach) and sciatic nerve block (parasacral approach).

On the ultrasound display, ultrasound views with and without the ultrasound generated by the photoacoustic effect were displayed during the procedure. In the study, the ultrasound generated by the photoacoustic effect was shown in white to prevent information bias.

The side showing ultrasound generated by the photoacoustic effect was blinded to the expert with an opacity board. Then, the expert performed the nerve block watching with a typical ultrasound view, then both of the movies were recorded. To confirm whether the nerve blocks were successful, 10 ml of acrylic paint solution was injected instead of a local anesthetic agent, and dissection performed after the experiment.

### Survey to evaluate needle tip visibility

Needle tip visibility was evaluated by a survey of participating anesthesiologists acting as volunteers. Recruitment of participants was performed through the local community of anesthesiologists, including department colleagues and anesthesiologists in associated hospitals. Exclusion criteria was rejection to participate in the survey. The authors and collaborators of the study were excluded from participating in the survey.

Ultrasound movies were randomly selected using a random number table. Participating anesthesiologists watched the ultrasound images and evaluated needle tip visibility in each movie using a Likert scale (score 1: very poor, 2: poor, 3: fair, 4: good, 5: very good). Demographic data of participants were also collected, including overall clinical experience and experience with ultrasound-guided nerve block. The survey was conducted on the Internet using questions posted with an invitation limit. Informed consent was obtained from all participants prior to the Internet questions. The primary outcome of the study was evaluation of needle tip visibility with or without the photoacoustic ultrasound. The secondary outcome was a relationship between experience with ultrasound-guided nerve block and needle tip visibility with or without the photoacoustic ultrasound.

### Statistical analysis

Likert scale scores from participant responses are expressed as median [first quartile, third quartile]. Clinical experience of participants is expressed in four rank groups (1–5, 6–10, 11–15, > 15 years) and the number of ultrasound-guided nerve block procedures expressed in four rank groups (0–50, 51–100, 101–200, > 200). Wilcoxon matched-pairs signed rank test was used to evaluate the scores. Spearman’s correlation coefficients (*rS*) were used to evaluate strength of associations among the variables. A preliminary study for estimating power analysis was performed using the authors and collaborators. The median score and standard deviation of photoacoustic ultrasound were expected to be superior in one score than without photoacoustic ultrasound. The sample size required for 80% power at ɑ = 0.05 was estimated to be sixteen participants. A p-value less than 0.05 was considered statistically significant. Statistical analyses were performed with GraphPad Prism, version 7.02 (GraphPad Software Inc., San Diego, USA).

## Results

**Figure 3:**
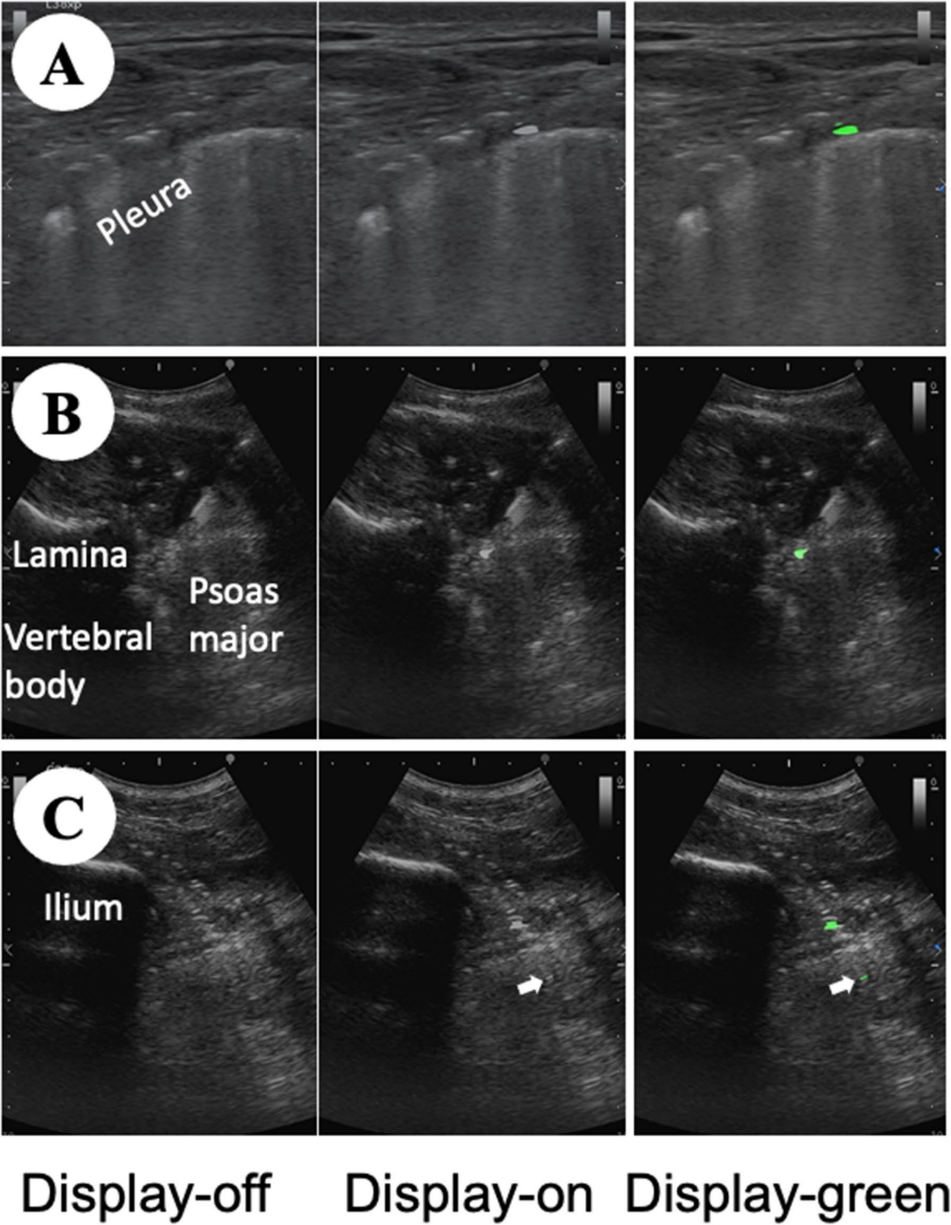
Ultrasound views of nerve blocks. The pictures show nerve blocks (at right side of cadaver). These pictures are all obtained using the photoacoustic needle. The picture of display-off does not show the photoacoustic ultrasound, the pictures of display-on and display-green show the photoacoustic ultrasound. Mirror image artifact of the needle tip is observed in the display-on and display-green of the (**C**) (white arrows indicate the mirror image artifact). (**A**) paravertebral block (**A**), (**B**) lumbar plexus block, (**C**) sciatic nerve block, display-off: no photoacoustic ultrasound, display-on: photoacoustic ultrasound using white color, display-green: photoacoustic ultrasound using green color (See Supplemental Digital Contents nos. 13, 15 and 17).

Twelve ultrasound movies were recorded (See Supplemental Digital Contents nos. 1–12, Fig. 3: sample scenes of the movies). Each nerve block was recorded using two types of movies, with or without the photoacoustic ultrasound. One of the movies was displayed on the photoacoustic ultrasound (display-on), but the other movie was displayed off of the photoacoustic ultrasound (display-off).

We invited forty anesthesiologists to participate in the study. Thirty anesthesiologists participated in this study with no exclusions. The clinical experience of participants in each group; 1–5, 6–10, 11–15, and > 15 years of experience were 11 (37%), 6 (20%), 7 (23%), and 6 (20%), respectively. The numbers of ultrasound-guided nerve block procedures performed by participants in each group; 0–50, 51–100, 101–200, and > 200 procedures were 12 (40%), 7 (23%), 2 (7%), and 9 (30%), respectively.

**Figure 4:**
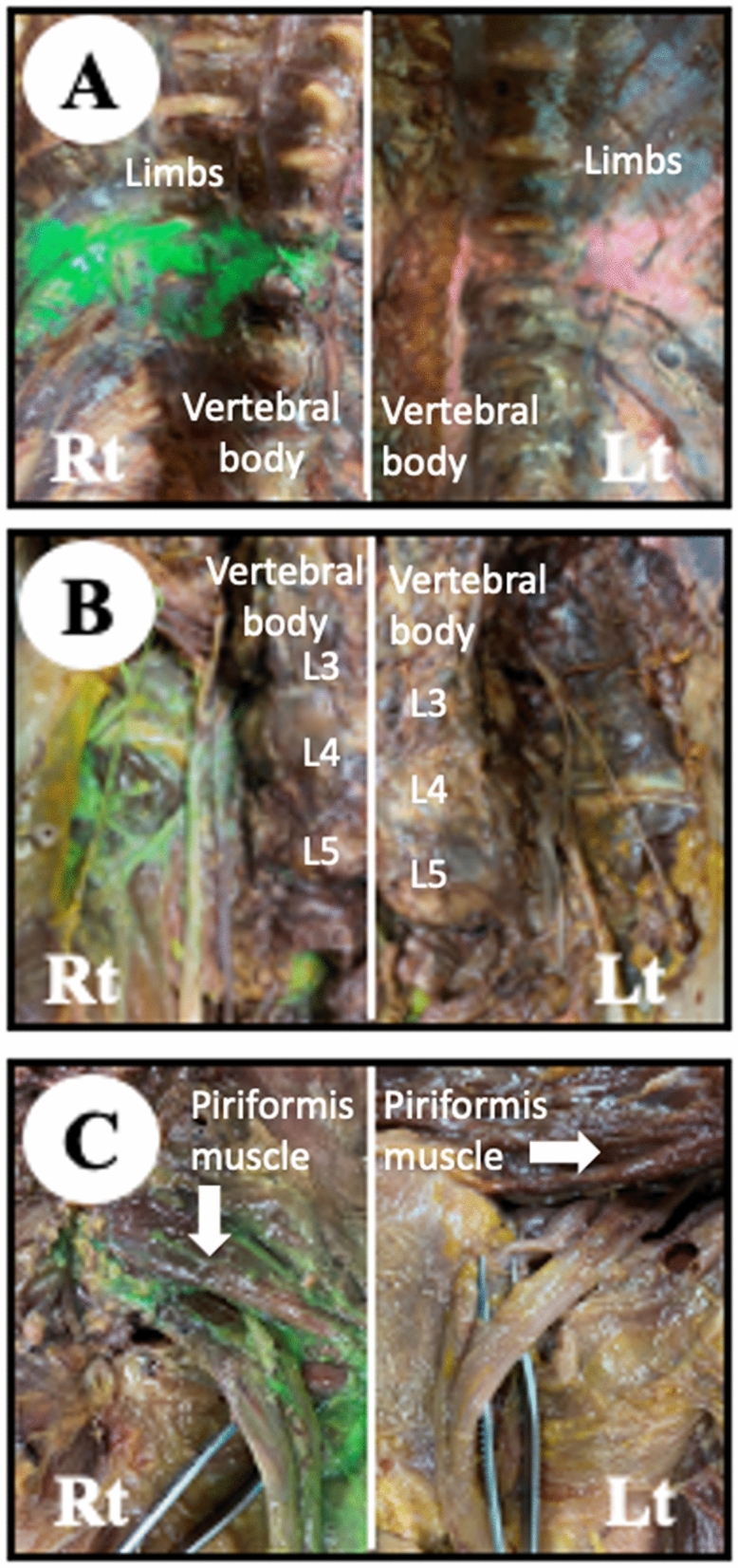
Dissected region in the cadaver. Paint injected during the deep nerve block at right- or left-side of a cadaver are green or pink, respectively. Pink stain at the left-side lumbar plexus block (**B**. Lt) and sciatic nerve block (**C**, Lt) are undetermined due to weak and blurred stain in connective tissue over the nerves. (**A**) Paravertebral block, (**B**) lumbar plexus block, (**C**) sciatic nerve block. *Rt* right-side of the cadaver, *Lt* left-side of the cadaver.

Needle trajectories of the deep nerve blocks were 35–40° in the paravertebral block (transverse technique, in-plane approach), and 60° in the lumbar plexus block (transverse technique, in-plane approach) or sciatic nerve block (parasacral approach). Dissection of the cadaver was performed to evaluate the quality of the nerve blocks. Most of the nerve blocks were confirmed as being successful. However, success of the left lumbar plexus block and the left sciatic nerve block could not be confirmed due to loss of paint (Fig. 4).

**Figure 5:**
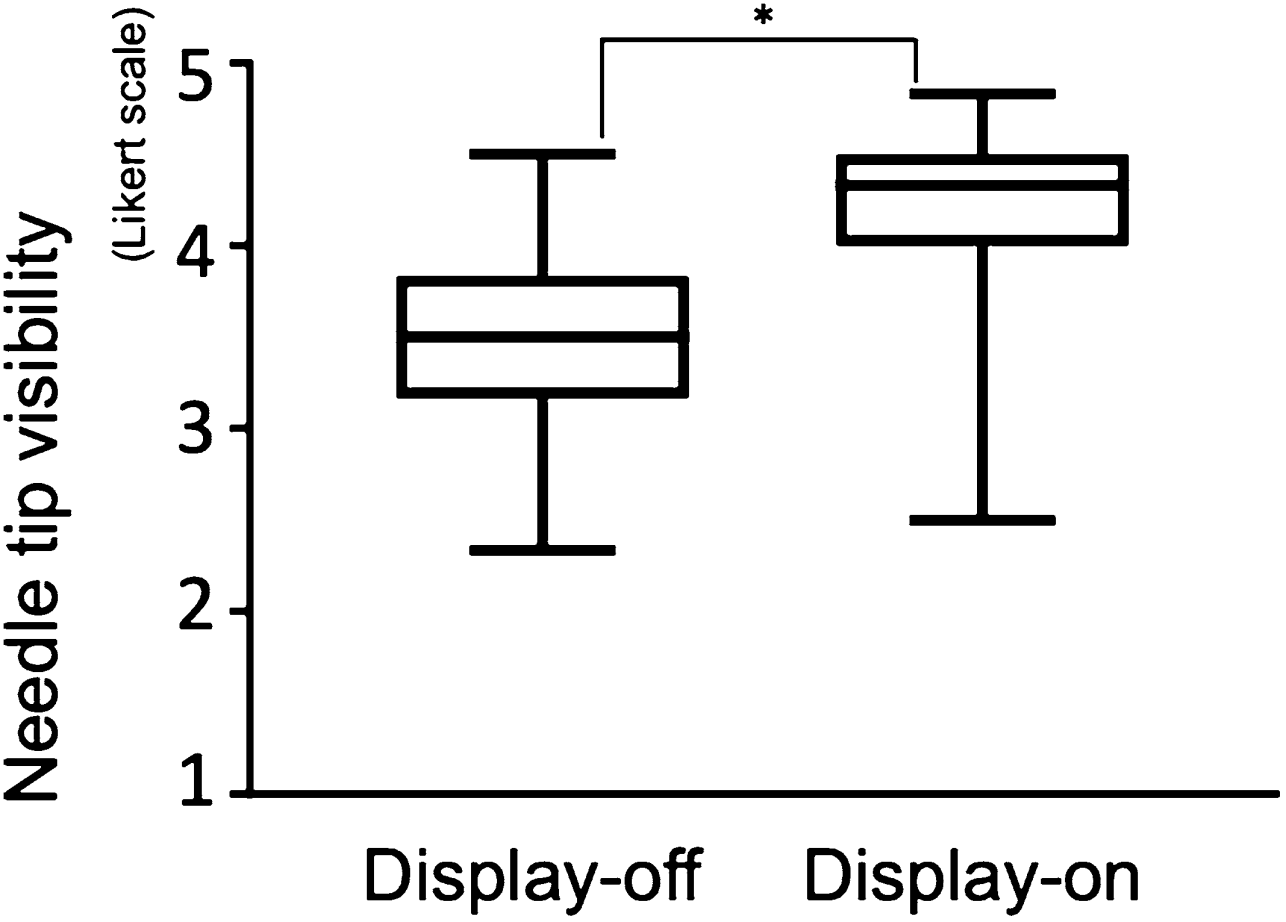
Needle tip visibility. The asterisk indicates a statistically significant difference between the display-off and display-on groups.

The Likert scale score for needle tip visibility in the display-on movie (4.3 [4.0, 4.5]) was significantly higher than that in the display-off movie (3.5 [3.2, 3.8]) (p < 0.01) (Fig. 5). Subgroup analysis of each nerve block had the same tendency (the paravertebral nerve block; display-on movie 4.5 [4.0, 4.5], display-off movie 3.5 [3.0, 4.0] (p < 0.01), the lumbar plexus block; display-on movie 4.5 [4.0, 5.0], display-off movie 4.0 [3.5, 4.5] (p = 0.01), the sciatic nerve block; display-on movie 4.0 [3.9, 4.5], display-off movie 3.3 [3.0, 3.6] (p < 0.01).

Correlation was shown between experience with ultrasound-guided nerve block and the score of needle tip visibility in the display-off movie (*rS* 0.43, p = 0.02). There was no correlation between other outcomes or the demographics of the participants.

## Discussion

This study demonstrates that a novel needle with an ultrasound-emitting needle-tip using the photoacoustic effect resulted in significantly improved visibility of the needle tip while performing simulated deep nerve blocks on a cadaver model. However, the difference in needle tip visibility scores between the display-on and display-off movies was only a median of 0.8, lower than that expected. The needle-tip was colored white on the ultrasound to prevent information bias. If we colored the needle-tip green instead of white during the study, it may greatly improve needle tip visibility. The visibility of the ultrasound-emitting needle tip is not dependant on sound waves reflected from the needle tip so a steeper angle of trajectory should not reduce tip visibility because ultrasound is emitted from the needle tip itself.

Commonly used needles for nerve blocks have a tendency to have decreased needle tip visibility at steeper trajectories. Several types of echogenic needles have been developed to improve needle tip visibility^10^, which may increase safety and efficacy^11^. Studies have shown that the visibility of an echogenic needle tip at a trajectory of 60° in a simulated environment is effective^2,3^. However, these ex-vivo results are difficult to translate directly to clinical practice^10^. The echogenic needles were not easily visible during ultrasound-guided injection in cadaver models with a needle trajectory greater than 60°^12^. Uultrasound beam steering increases needle visibility^3^. However, the beam steering dose not improve needle visiblity at a 70° trajectory angle^3^. The visibility of the photoacoustic needle tip is effective at a needle angle of 80° (Fig. 2 and “Supplemental Digital Contents”). We suggest that the clinical efficacy of a photoacoustic needle may facilitate performing ultrasound-guided deep nerve blocks, especially using the out-of-plane technique^13^.

**Figure 6:**
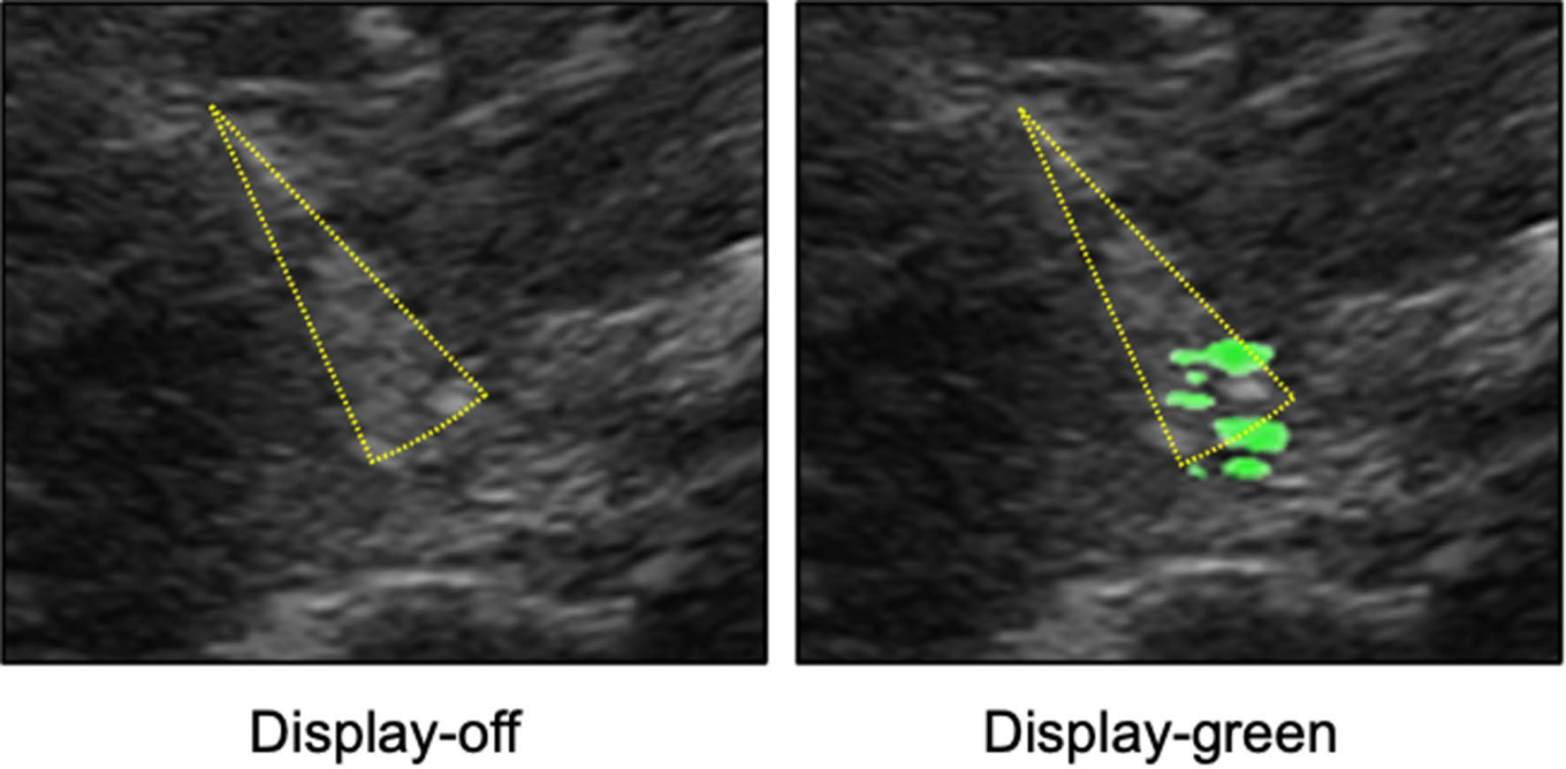
Artifacts cause multiple needle tips. Sciatic nerve block on the left side of the cadaver (See Supplemental Digital Content nos. 11 and 18). Combination of reverberation artifact and bayonet artifact (dotted yellow triangle) may cause an illusion of multiple needle tips (display-green; Supplemental Digital Content no. 18). The needle tip without displaying photoacoustic ultrasound cannot be identified (display-off; Supplemental Digital Content no. 11). Display-off: no photoacoustic ultrasound, display-green: photoacoustic ultrasound using green color.

Visualization of the needle during insertion is a basic skill during ultrasound-guided peripheral nerve block^14^. The training needed to achieve skill in needle tip visualization varies among trainees^15^. In the study, correlation was found between experience in ultrasound-guided nerve block and the scores of needle tip visibility in the display-off movie. In another words, participants with more extensive experience in nerve blocks tended to appreciate conventional echogenic needles. In the display-on movie, high-power ultrasound from the needle tip may cause a reverberation artifact^16^, which induces an illusion of multiple needle tips (Fig. 6). That artifact may induce discomfort in participants with greater experience in nerve blocks. We think that anesthesiologists may overcome pitfalls induced by these artifacts by understanding details of adjusting the appropriate ultrasound gain and physical characteristics of ultrasound.

In the system, the ultrasound frame rate is reduced due to sharing frames for detecting photoacoustic ultrasound. The frame rate constructing the ultrasound view keeps 89% of the total frame rate, which may not reduce image quality and add no stress to the operator during the nerve block without sensing time lag. Hence, we believe that the reduced frame rate of 11% may not influence in the study result.

Needle tip visibility in tissues in a live human was reported to be less than that in cadaveric tissue^17^. Hence, there is still a question whether good visibility of the photoacoustic needle tip can be achieved a live human. This is a limitation of this study. Lediju Bell and Shubert have demonstrated good visibility of photoacoustic needles in fat and other tissues^18,19^. These results suggest the efficacy of photoacoustic needles in obese patients*.* Photoacoustic ultrasound in clinical use has been a subject of very considerable interest during the past few years. We believe that this innovation will resolve technical problems associated with deep nerve blocks.

## Conclusion

Ultrasound-emitted from a needle tip using the photoacoustic effect improved needle tip visibility during deep nerve blocks in a cadaver model.

## Supplementary Information

Supplementary Information 1.

Supplementary Video 1.

Supplementary Video 2.

Supplementary Video 3.

Supplementary Video 4.

Supplementary Video 5.

Supplementary Video 6.

Supplementary Video 7.

Supplementary Video 8.

Supplementary Video 9.

Supplementary Video 10.

Supplementary Video 11.

Supplementary Video 12.

Supplementary Video 13.

Supplementary Video 14.

Supplementary Video 15.

Supplementary Video 16.

Supplementary Video 17.

Supplementary Video 18.
