# Supplementary material for: Photoacoustic needle improves needle tip visibility during deep peripheral nerve block
Source: Sci Rep. 2021 Apr 19;11:8432. doi: 10.1038/s41598-021-87777-9 (PMC8055898; doi:10.1038/s41598-021-87777-9)
Supplement: Supplementary file 1 — Supplementary Information 1. [file 41598_2021_87777_MOESM1_ESM.doc]

**Photoacoustic needle improves needle tip visibility during deep peripheral nerve block**

Kunitaro Watanabe, Joho Tokumine, Alan Kawarai Lefor, Harumasa Nakazawa, Katsuya Yamamoto, Hiroyuki Karasawa, Miki Nagase, Tomoko Yorozu

**CONSORT Diagram**

**Analysis**

**Enrollment**

**Allocation**

**Follow-Up**

Recruiting participants (n=40)

Excluded (n=10)

♦  Not meeting inclusion criteria (n=0)

♦  Declined to participate (n=10)

♦  Other reasons (n=0)

Analysed the questionnaire of the participants (n=30)
♦ Excluded from analysis (n=0)

All participants replied back the questionnaire (n=30).

Lost to follow-up (n=0)

Discontinued intervention (n=0)

Twelve ultrasound movies were randomly allocated using a random number table.

Participants watched the movies and evaluated needle tip visibility (Likert scale 1-5)

♦ Received allocated intervention (n=30)

♦ Did not receive allocated intervention (n=0)
